# Patent Medicines

**Published:** 1871-04

**Authors:** 


### PATENT MEDICINES

Are those remedies for sickness and disease whose constituents are unknown, except to the maker of them, who is said to be the "proprietor." There is really no "patent" medicine in the United States, in the sense in which that expression is used; for, in the first place, the getting a "patent" for it at Washington would reveal its constituents, and the public would soon know that cough medicines were nothing more than the various preparations of morphia, and that the "Universal Disease Eradicator" was nothing but bread pills made up with the tincture of T(hy)ime; that is, if you are sick, take time, wait awhile, and you will get better, in very many cases indeed.

But "patent," as applied to medicines, ought to come from some word which means to cover, to hide, to keep secret; they are secret medicines; secret, because their constituents are not made public. That all such medicines do immeasurably more harm than good, can scarcely be denied. Many of them are perfectly inert; but while the misguided taker of it is waiting for its good effects, the disease may be spreading in the body and eating out its life.

If, on the other hand, a patent medicine is really efficient, it is liable, as some of them certainly are, to place the system in a condition which impels their use for a life-time; or, in some other cases, poison it by slow degrees, and eventually destroys life.

It may be safe to say that any man who habitually takes any medicine, the constituents of which are unknown to him or his medical adviser, acts most unwisely, and endangers his own life.

The French are an age or two ahead of us in this respect. No man is allowed, under very severe penalties, to sell any medicine without having all the constituents and their proportions printed on what contains it. New York State, after the pattern of Illinois, has under consideration a similar law, and it is to be hoped that it will not fail of enactment. The sale of patent medicines is the greatest cruelty and the greatest swindle of the age; for patronized, as they mainly are, by the poor, who are not able to pay for competent medical advice, and are not disposed to apply to a hospital or dispensary for free aid, are the chief sufferers, because the effect is as if they threw their hard earnings into the sea, for the reasons above named.

It is not by any means claimed that patent medicines never do any good, for in reality most of them are the prescriptions of eminent medical men, and were adapted to special cases; but to apply them indiscriminately to a variety of forms of disease, and to do it for the sake of a little money, when such great risks are run as have already been referred to, merits the indignation and contempt of every intelligent mind. "Give me a dollar for my medicine or die," is the summing up of the character of every man who sells a secret cure for disease.

